# Supplementary material for: Genome-Wide Analysis of the RAV Gene Family in Wheat and Functional Identification of TaRAV1 in Salt Stress
Source: Int J Mol Sci. 2022 Aug 9;23(16):8834. doi: 10.3390/ijms23168834 (PMC9408559; doi:10.3390/ijms23168834)
Supplement: Supplementary file 1 [file ijms-23-08834-s001.zip › Figure S1.pdf]

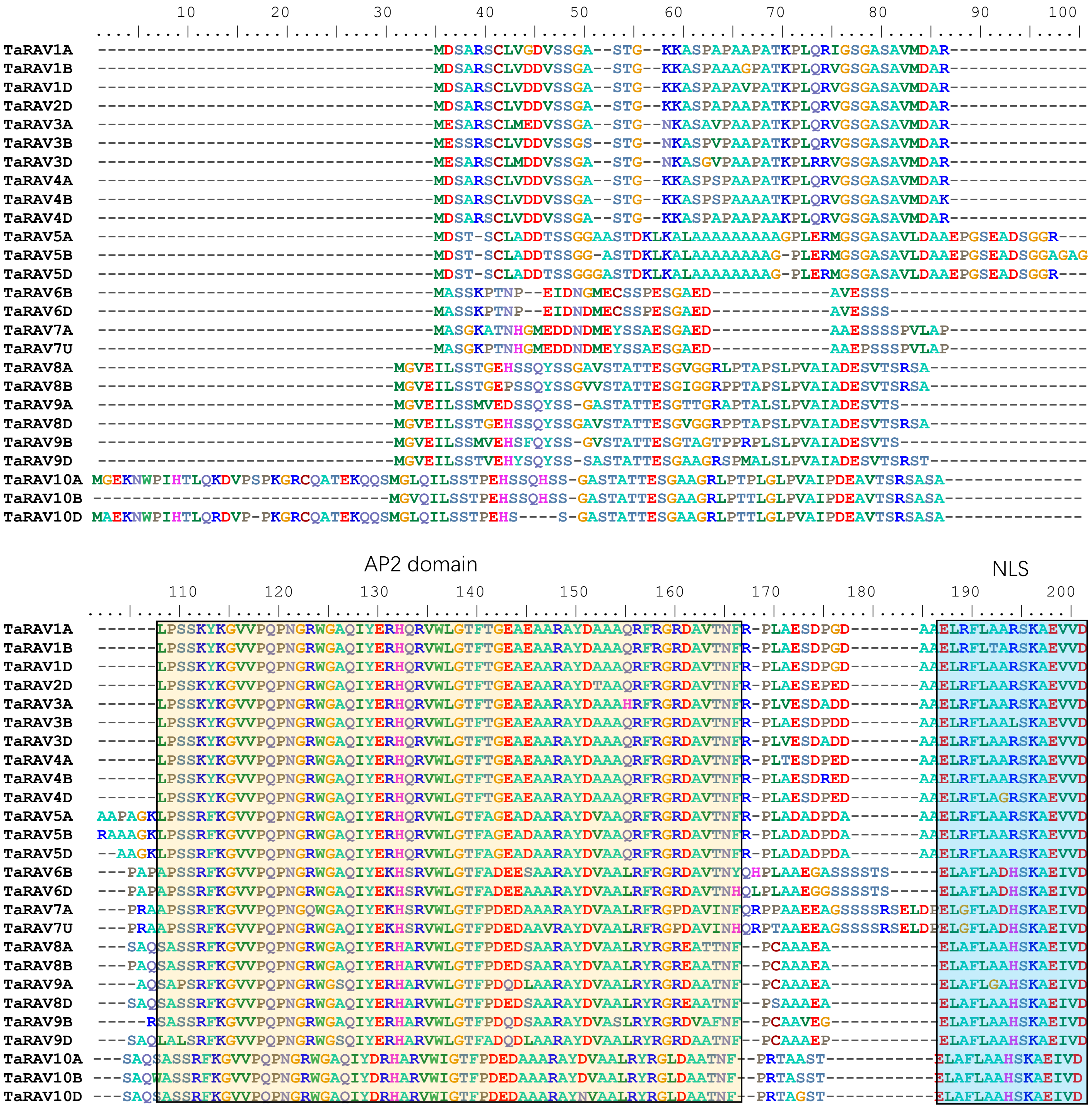
AP2 domain
NLS
TaRAV1A
TaRAV1B
TaRAV1D
TaRAV2D
TaRAV3A
TaRAV3B
TaRAV3D
TaRAV4A
TaRAV4B
TaRAV4D
TaRAV5A
TaRAV5B
TaRAV5D
TaRAV6B
TaRAV6D
TaRAV7A
TaRAV7U
TaRAV8A
TaRAV8B
TaRAV9A
TaRAV8D
TaRAV9B
TaRAV9D
TaRAV10A
TaRAV10B
TaRAV10D

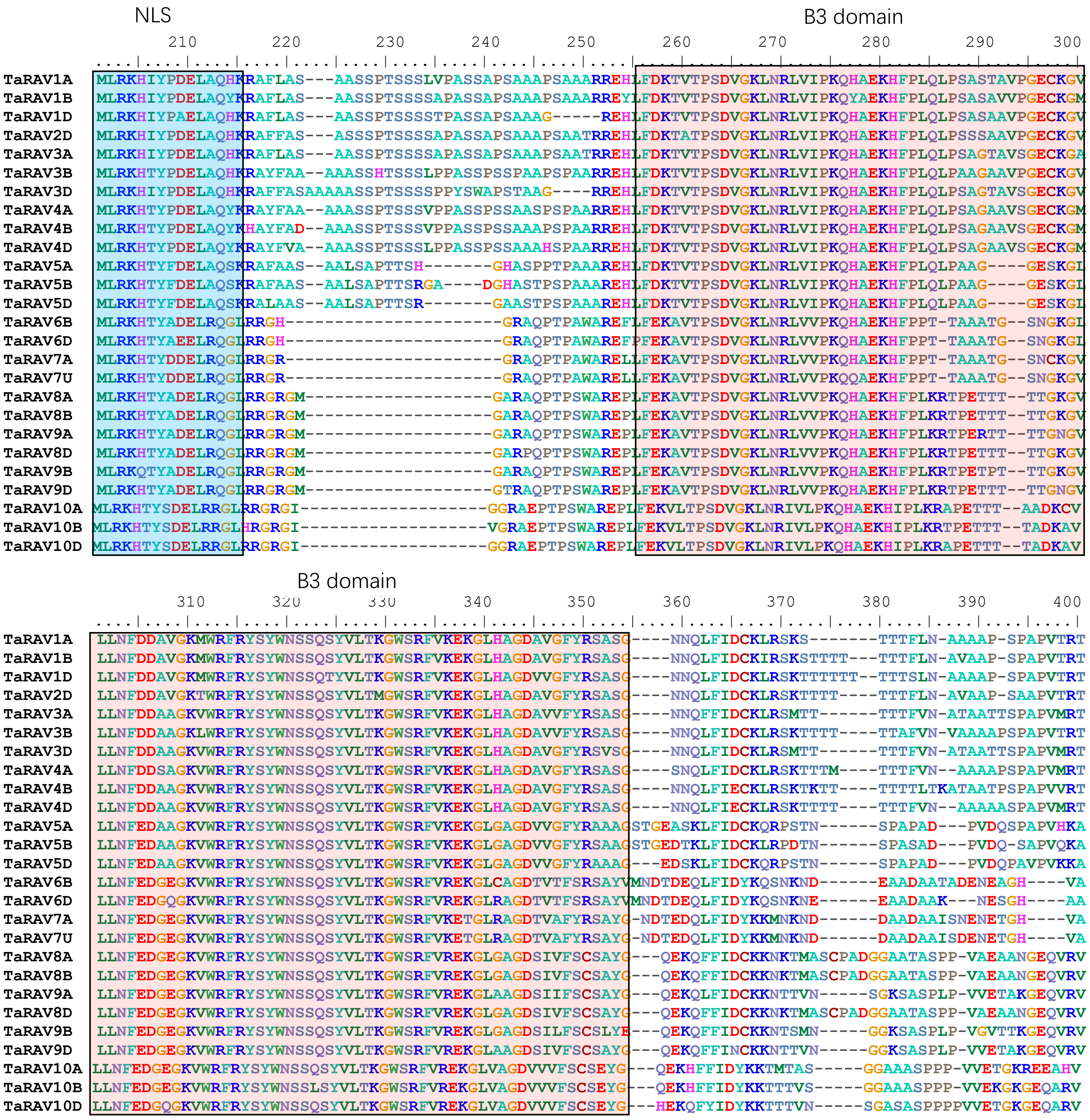
NLS
B3 domain
210 220 230 240 250 260 270 280 290 300
TaRAV1A MLRKHIYPDELAQHKRAFLAS---AASSPTSSSLVPASSAPSAAAPSAAARREHLFDKTVTPSDVGKLNRLVIPKQHAEKHFPLQLPSASTAVPGECKGV
TaRAV1B MLRKHIYPDELAQYKRAFLAS---AASSPTSSSSAPASSAPSAAAPSAAARREYLFDKTVTPSDVGKLNRLVIPKQYAEKHFPLQLPSASAVVPGECKGM
TaRAV1D MLRKHIYPAELAQHKRAFLAS---AASSPTSSSSTPASSAPSAAAG-----REHLFDKTVTPSDVGKLNRLVIPKQHAEKHFPLQLPSASAAVPGECKGV
TaRAV2D MLRKHIYPDELAQHKRAFFAS---ASSSPTSSSSAPASSAPSAAAPSAATRREHLFDKTATPSDVGKLNRLVIPKQHAEKHFPLQLPSSSAAVPGECKGV
TaRAV3A MLRKHIYPDELAQHKRAFLAS---AASSPTSSSSAPASSAPSAAAPSAATRREHLFDKTVTPSDVGKLNRLVIPKQHAEKHFPLQLPSAGTAVSGECKGA
TaRAV3B MLRKHTYPDELAQHKRAYFAA--AAASSHTSSSLPPASSPSSPAAPSPAARREHLFDKTVTPSDVGKLNRLVIPKQHAEKHFPLQLPAAGAAVPGECKGV
TaRAV3D MLRKHIYPDELAQHKRAFFASAAAAASSPTSSSSPPYSWAPSTAAG----RREHLFDKTVTPSDVGKLNRLVIPKQHAEKHFPLQLPSAGTAVSGECKGV
TaRAV4A MLRKHTYPDELAQYKRAYFAA--AAASSPTSSSVPPASSPSSAASPSPAARREHLFDKTVTPSDVGKLNRLVIPKQHAEKHFPLQLPSAGAAVSGECKGM
TaRAV4B MLRKHTYPDELAQYKHAYFAD--AAASSPTSSSVPPASSPSSAASPSPAARREHLFDKTVTPSDVGKLNRLVIPKQHAEKHFPLQLPSAGAAVSGECKGM
TaRAV4D MLRKHTYPDELAQYKRAYFVA--AAASSPTSSSLPPASSPSSAAAHSPAARREHLFDKTVTPSDVGKLNRLVIPKQHAEKHFPLQLPSAGAAVSGECKGM
TaRAV5A MLRKHTYFDELAQSKRAFAAS--AALSAPTTSH-------GHASPPTPAAAREHLFDKTVTPSDVGKLNRLVIPKQHAEKHFPLQLPAAG----GESKGL
TaRAV5B MLRKHTYFDELAQSKRAFAAS--AALSAPTTSRGA----DGHASTPSPAAAREHLFDKTVTPSDVGKLNRLVIPKQHAEKHFPLQLPAAG----GESKGL
TaRAV5D MLRKHTYFDELAQSKRALAAS--AALSAPTTSR-------GAASTPSPAAAREHLFDKTVTPSDVGKLNRLVIPKQHAEKHFPLQLPAAG----GESKGL
TaRAV6B MLRKHTYADELRQGLRRGH----------------------GRAQPTPAWAREFLFEKAVTPSDVGKLNRLVVPKQHAEKHFPPT-TAAATG--SNGKGL
TaRAV6D MLRKHTYAEELRQGLRRGH----------------------GRAQPTPAWAREFPFEKAVTPSDVGKLNRLVVPKQHAEKHFPPT-TAAATG--SNGKGL
TaRAV7A MLRKHTYDDELRQGLRRGR----------------------GRAQPTPAWARELLFEKAVTPSDVGKLNRLVVPKQHAEKHFPPT-TAAATG--SNCKGV
TaRAV7U MLRKHTYDDELRQGLRRGR----------------------GRAQPTPAWARELLFEKAVTPSDVGKLNRLVVPKQQAEKHFPPT-TAAATG--SNGKGV
TaRAV8A MLRKHTYADELRQGLRRGRGM-------------------GARAQPTPSWAREPLFEKAVTPSDVGKLNRLVVPKQHAEKHFPLKRTPETTT--TTGKGV
TaRAV8B MLRKHTYADELRQGLRRGRGM-------------------GARAQPTPSWAREPLFEKAVTPSDVGKLNRLVVPKQHAEKHFPLKRTPETTT--TTGKGV
TaRAV9A MLRKHTYADELRQGLRRGRGM-------------------GARAQPTPSWAREPLFEKAVTPSDVGKLNRLVVPKQHAEKHFPLKRTPERTT--TTGNGV
TaRAV8D MLRKHTYADELRQGLRRGRGM-------------------GARPQPTPSWAREPLFEKAVTPSDVGKLNRLVVPKQHAEKHFPLKRTPETTT--TTGKGV
TaRAV9B MLRKQTYADELRQGLRRGRGM-------------------GARAQPTPSWAREPLFEKAVTPSDVGKLNRLVVPKQHAEKHFPLKRTPETPT--TTGKGV
TaRAV9D MLRKHTYADELRQGLRRGRGM-------------------GTRAQPTPSWAREPLFEKAVTPSDVGKLNRLVVPKQHAEKHFPLKRTPETTT--TTGNGV
TaRAV10A MLRKHTYSDELRRGLRRGRGI-------------------GGRAEPTPSWAREPLFEKVLTPSDVGKLNRIVLPKQHAEKHIPLKRAPETTT--AADKCV
TaRAV10B MLRKHTYSDELRRGLHRGRGI-------------------VGRAEPTPSWAREPLFEKVLTPSDVGKLNRIVLPKQHAEKHIPLKRTPETTT--TADKAV
TaRAV10D MLRKHTYSDELRRGLRRGRGI-------------------GGRAEPTPSWAREPLFEKVLTPSDVGKLNRIVLPKQHAEKHIPLKRAPETTT--TADKAV
B3 domain
310 320 330 340 350 360 370 380 390 400
TaRAV1A LLNFDDAVGKMWRFRYSYWNSSQSYVLTKGWSRFVKEKGLHAGDAVGFYRSASG----NNQLFIDCKLRSKS-------TTTFLN-AAAAP-SPAPVTRT
TaRAV1B LLNFDDAVGKMWRFRYSYWNSSQSYVLTKGWSRFVKEKGLHAGDAVGFYRSASG----NNQLFIDCKIRSKSTTTT---TTTFLN-AVAAP-SPAPVTRT
TaRAV1D LLNFDDAVGKMWRFRYSYWNSSQTYVLTKGWSRFVKEKGLHAGDVVGFYRSASG----NNQLFIDCKLRSKTTTTTT--TTTSLN-AAAAP-SPAPVTRT
TaRAV2D LLNFDDAVGKTWRFRYSYWNSSQSYVLTMGWSRFVKEKGLHAGDAVGFYRSASG----NNQLFIDCKLRSKTTTT----TTTFLN-AVAAP-SAAPVTRT
TaRAV3A LLNFDDAAGKVWRFRYSYWNSSQSYVLTKGWSRFVKEKGLHAGDAVVFYRSASG----NNQFFIDCKLRSMTT------TTTFVN-ATAATTSPAPVMRT
TaRAV3B LLNFDDAAGKLWRFRYSYWNSSQSYVLTKGWSRFVKEKGLHAGDAVVFYRSASG----NNQLFIDCKLRSKTTTT----TTAFVN-VAAAAPSPAPVTRT
TaRAV3D LLNFDDAAGKVWRFRYSYWNSSQSYVLTKGWSRFVKEKGLHAGDAVGFYRSVSG----NNQLFIDCKLRSMTT------TTTFVN-ATAATTSPAPVMRT
TaRAV4A LLNFDDSAGKVWRFRYSYWNSSQSYVLTKGWSRFVKEKGLHAGDAVGFYRSASG----SNQLFIDCKLRSKTTTM----TTTFVN--AAAAPSPAPVMRT
TaRAV4B LLNFDDAAGKVWRFRYSYWNSSQSYVLTKGWSRFVKEKGLHAGDAVGFYRSASG----NNQLFIECKLRSKTKTT----TTTTLTKATAATPSPAPVVRT
TaRAV4D LLNFDDAAGKVWRFRYSYWNSSQSYVLTKGWSRFVKEKGLHAGDAVGFYRSASG----NNQLFIECKLRSKTTTT----TTTFVN--AAAAASPAPVMRT
TaRAV5A LLNFEDAAGKVWRFRYSYWNSSQSYVLTKGWSRFVKEKGLGAGDVVGFYRAAAGSTGEASKLFIDCKQRPSTN------SPAPAD---PVDQSPAPVHKA
TaRAV5B LLNFEDAAGKVWRFRYSYWNSSQSYVLTKGWSRFVKEKGLGAGDVVGFYRSAAGSTGEDTKLFIDCKLRPDTN------SPASAD---PVDQ-SAPVQKA
TaRAV5D LLNFEDAAGKVWRFRYSYWNSSQSYVLTKGWSRFVKEKGLGAGDVVGFYRAAAG---EDSKLFIDCKQRPSTN------SPAPAD---PVDQPAVPVKKA
TaRAV6B LLNFEDGEGKVWRFRYSYWNSSQSYVLTKGWSRFVREKGLCAGDTVTFSRSAYVMNDTDEQLFIDYKQSNKND------EAADAATADENEAGH----VA
TaRAV6D LLNFEDGQGKVWRFRYSYWNSSQSYVLTKGWSRFVREKGLRAGDTVTFSRSAYVMNDTDEQLFIDYKQSNKNE------EAADAAK---NESGH----AA
TaRAV7A LLNFEDGEGKVWRFRYSYWNSSQSYVLTKGWSRFVKETGLRAGDTVAFYRSAYG-NDTEDQLFIDYKKMNKND------DAADAAISNENETGH----VA
TaRAV7U LLNFEDGEGKVWRFRYSYWNSSQSYVLTKGWSRFVKETGLRAGDTVAFYRSAYG-NDTEDQLFIDYKKMNKND------DAADAAISDENETGH----VA
TaRAV8A LLNFEDGEGKVWRFRYSYWNSSQSYVLTKGWSRFVREKGLGAGDSIVFSCSAYG---QEKQFFIDCKKNKTMASCPADGGAATASPP-VAEAANGEQVRV
TaRAV8B LLNFEDGEGKVWRFRYSYWNSSQSYVLTKGWSRFVREKGLGAGDSIVFSCSAYG---QEKQFFIDCKKNKTMASCPADGGAATASPP-VAEAANGEQVRV
TaRAV9A LLNFEDGEGKVWRFRYSYWNSSQSYVLTKGWSRFVREKGLAAGDSIIFSCSAYG---QEKQLFIDCKKNTTVN-----SGKSASPLP-VVETAKGEQVRV
TaRAV8D LLNFEDGEGKVWRFRYSYWNSSQSYVLTKGWSRFVREKGLGAGDSIVFSCSAYG---QEKQFFIDCKKNKTMASCPADGGAATASPP-VAEAANGEQVRV
TaRAV9B LLNFEDGEGKVWRFRYSYWNSSQSYVLTKGWSRFVREKGLGAGDSILFSCSLYE---QEKQFFIDCKKNTSMN-----GGKSASPLP-VGVTTKGEQVRV
TaRAV9D LLNFEDGEGKVWRFRYSYWNSSQSYVLTKGWSRFVREKGLAAGDSIVFSCSAYG---QEKQFFINCKKNTTVN-----GGKSASPLP-VVETAKGEQVRV
TaRAV10A LLNFEDGEGKVWRFRYSYWNSSQSYVLTKGWSRFVREKGLVAGDVVVFSCSEYG---QEKHFFIDYKKTMTAS-----GGAAASPPP-VVETGKREEAHV
TaRAV10B LLNFEDGEGKVWRFRYSYWNSSLSYVLTKGWSRFVREKGLVAGDVVVFSCSEYG---QEKHFFIDYKKTTTVS-----GGAAASPPP-VVEKGKGEQARV
TaRAV10D LLNFEDGQGKVWRFRYSYWNSSQSYVLTKGWSRFVREKGLVAGDVVVFSCSEYG---HEKQFYIDYKKTTTVN-----SGASASPPPPVVETGKGEQARV

```
                 410       420       430       440       450       460       470       480       490       500
          ....|....|....|....|....|....|....|....|....|....|....|....|....|....|....|....|....|....|....|....|
TaRAV1A   VRLFGVDLLTAP----APEHEEYGLVPKTNKR--SMDASVAAPTPAHAVWKKRCVDFALTYRLATTSQCPRSRDQVEGVQAAGSTFAL------------
TaRAV1B   VRLFGVDLLAAP----APEHEEYGMA-KTNKR--SMDASVEAPTPAHAVWKKRCVDFALTYRLATAPEFPRSRDQLEGVQAAGSTFAL------------
TaRAV1D   VRLFGVDLLTAP----APEQEEYGMA-KTNKR--SMDASVAAPTPAHAVWKKRCVDFALTYRHATTSQCPRPRDQLKGVQAAGCAFAL------------
TaRAV2D   VRLFGVDLPTAP----APEREEYGMT-KTNKK--SMDASVAAPTPVHAVWKKRCVDFALTYRHATTSQCPRSRDQLEGVQGAGSTFAL------------
TaRAV3A   VRLFGVDLLTAPALRHAPEHEDYGMA-KTNKR--FVDASVAAATPAHAVWKKRCVDFSLTYSHATTSQCPTSRDQLEGIQAAGSTFALQTCHHSPHSARG
TaRAV3B   VRLFGVDLLTAP----APEHEEYGMA-KTNKR--SMDASVEAPTPAHAVWKKRCVDFAL------IPQCPRSRDQLVGVQAAESTFAL------------
TaRAV3D   VRLFGVDLLTAPAPRHAPEHEDYGLA-KTNKRSVSVDASVAAAIPAHAVWKKRCVDFSLTYRLTTTPQCPRSGRRDELVQAAGSTFAL------------
TaRAV4A   VRLFGVDLLTAPAPSHAPEHEDCSMVPKTSKR--SMDAS-AAATPAHAVWKKRCIDFALT----------------------------------------
TaRAV4B   VRLFGVDLLTAPAPRHAPEHENYGMTKRS--R--DADV--AAPTPVHAVWKKRCMDFALT----------------------------------------
TaRAV4D   VRLFGVDLLTAPAPSHAPEHEDCSMVPKTYKR--SMDTS-AAPTPAHAVWKKQCIDFALT----------------------------------------
TaRAV5A   VRIFGVDLLTAP------ASP-----GCKRAR------DMVKPPAPTAALKKQCIELALA----------------------------------------
TaRAV5B   VRLFGVDLLTAP------ASPEQGMPGCKRAR------DLVKSPPPKVAFKKQCIELALA----------------------------------------
TaRAV5D   VRLFGVDLLTAP------ASPEQGMPGCKRAR------DLVKSPPPTAAFKKQCIELALA----------------------------------------
TaRAV6B   VKLFGVDIGG------------GGMGGSS-------------------------------------GG--------------------------------
TaRAV6D   VKLFGVDIGG------------GGMAGSS-------------------------------------GG--------------------------------
TaRAV7A   VKLFGVDIAG------------GGMVGSS-------------------------------------GG--------------------------------
TaRAV7U   VKLFGVDIAG------------GGMAGSS-------------------------------------GG--------------------------------
TaRAV8A   VRLFGVDIAGEK----------RGRVAQA----------------EQELFKRQCV--AHSQHSPALGAFVL-----------------------------
TaRAV8B   VRLFGVDIAGEK----------RGRAAPA----------------EQELFKRQCV--AHSQHSPALGAIVL-----------------------------
TaRAV9A   VRLFGVDIAGVK----------RGRAATA--E-----------QGPPELLKRQCVPLPHGQRSPALGAFVL-----------------------------
TaRAV8D   VRLFGVDIAGEK----------RGRAAPA----------------EQELFKRQCV--AHSQHSPALGAIVL-----------------------------
TaRAV9B   VRLFGVDISGVK----------RGRAATATAE-----------QGLQELFKRQCV--APGQHSPALGAFAL-----------------------------
TaRAV9D   VRLFGVDIAGVK----------RGRAATA--E-----------HGPPELFKRQCV--AHGQHSPALGAFVL-----------------------------
TaRAV10A  VRLFGVDLAGEM----------RGLAAPA----------------GLELFKRQWH---------------------------------------------
TaRAV10B  VRLFGVDLAGEM----------RGLAAPA----------------ELELFKRQ-----------------------------------------------
TaRAV10D  VRLFGVDLAGEM----------RGLAAPA----------------ELELFKRQ-----------------------------------------------
```

Figure S1. Alignment of the 26 RAV proteins in wheat.
