# Supplementary material for: Genome-Wide Analysis of the RAV Gene Family in Wheat and Functional Identification of TaRAV1 in Salt Stress
Source: Int J Mol Sci. 2022 Aug 9;23(16):8834. doi: 10.3390/ijms23168834 (PMC9408559; doi:10.3390/ijms23168834)
Supplement: Supplementary file 1 [file ijms-23-08834-s001.zip › Figure S2.pdf]

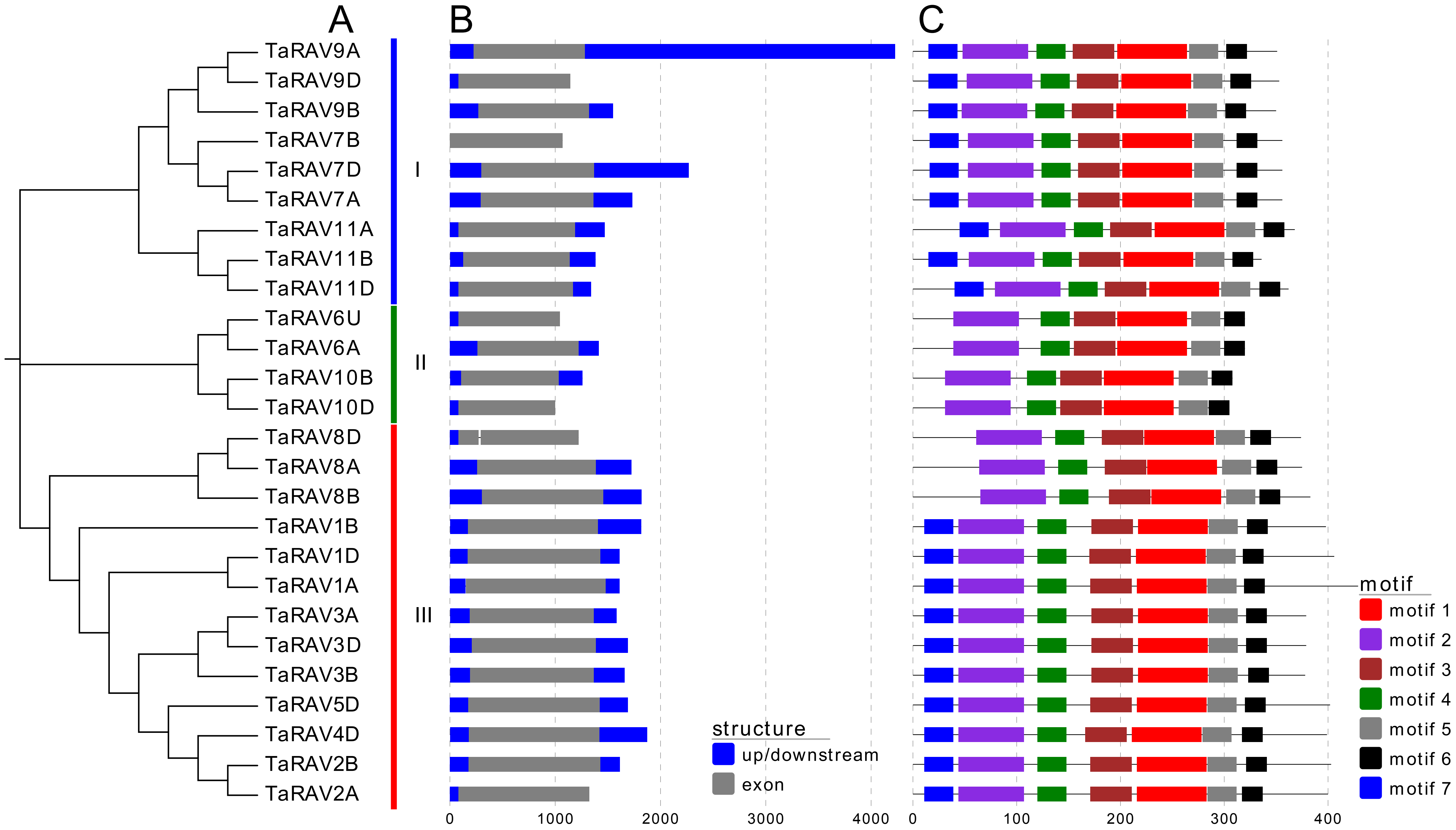

Supplementary Figure S2. Intron–exon structures and conserved motifs of the wheat RAV genes. The intron-exon structures were produced using the GSDS online tool. The exons are indicatedand by the gray boxes, the untranslated regions (UTRs) are indicatedand by the blue box, the introns are indicatedand by the black lines.
