# Supplementary material for: Genome-Wide Analysis of the RAV Gene Family in Wheat and Functional Identification of TaRAV1 in Salt Stress
Source: Int J Mol Sci. 2022 Aug 9;23(16):8834. doi: 10.3390/ijms23168834 (PMC9408559; doi:10.3390/ijms23168834)
Supplement: Supplementary file 1 [file ijms-23-08834-s001.zip › Figure S4.pdf]

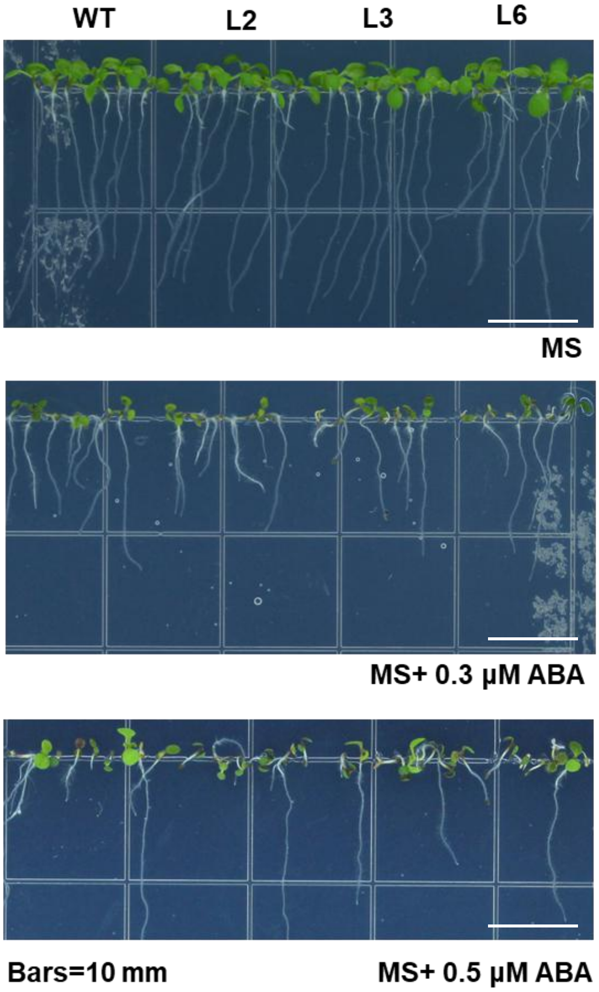

Figure S4. Phenotype of *TaRAV1* transgenic Arabidopsis grown on MS medium with or without ABA for 8 days.
