# Supplementary figures and images for: Genome-Wide Analysis of the RAV Gene Family in Wheat and Functional Identification of TaRAV1 in Salt Stress
Source: Int J Mol Sci. 2022 Aug 9;23(16):8834. doi: 10.3390/ijms23168834 (PMC9408559; doi:10.3390/ijms23168834)

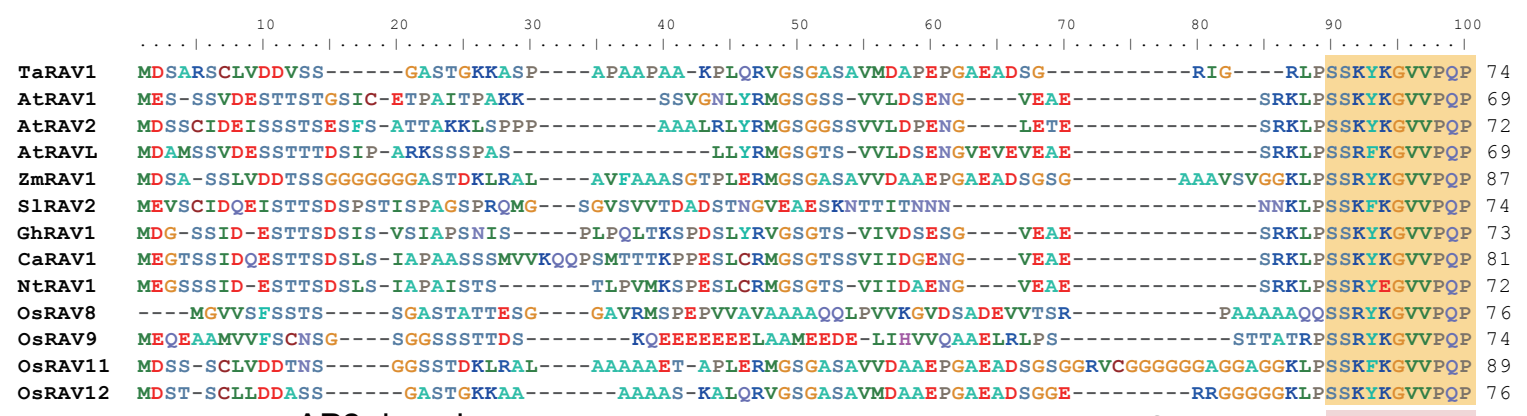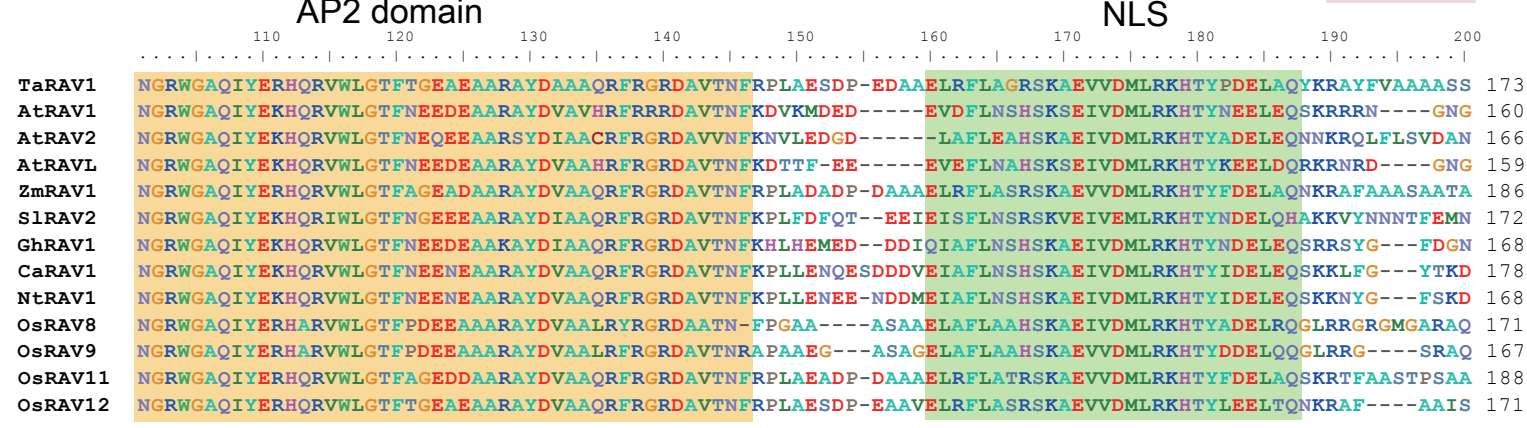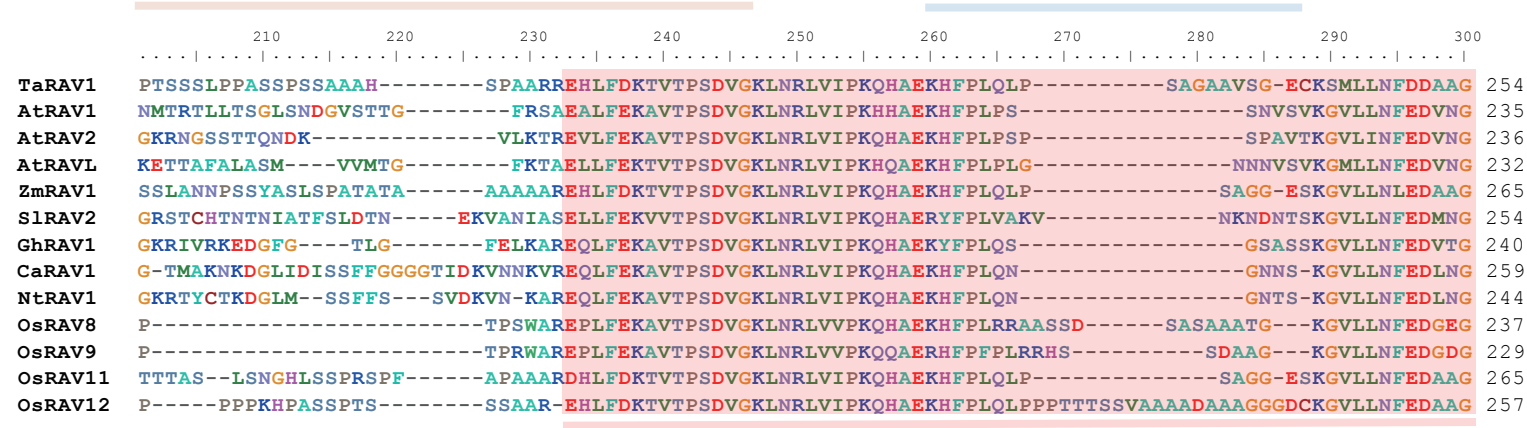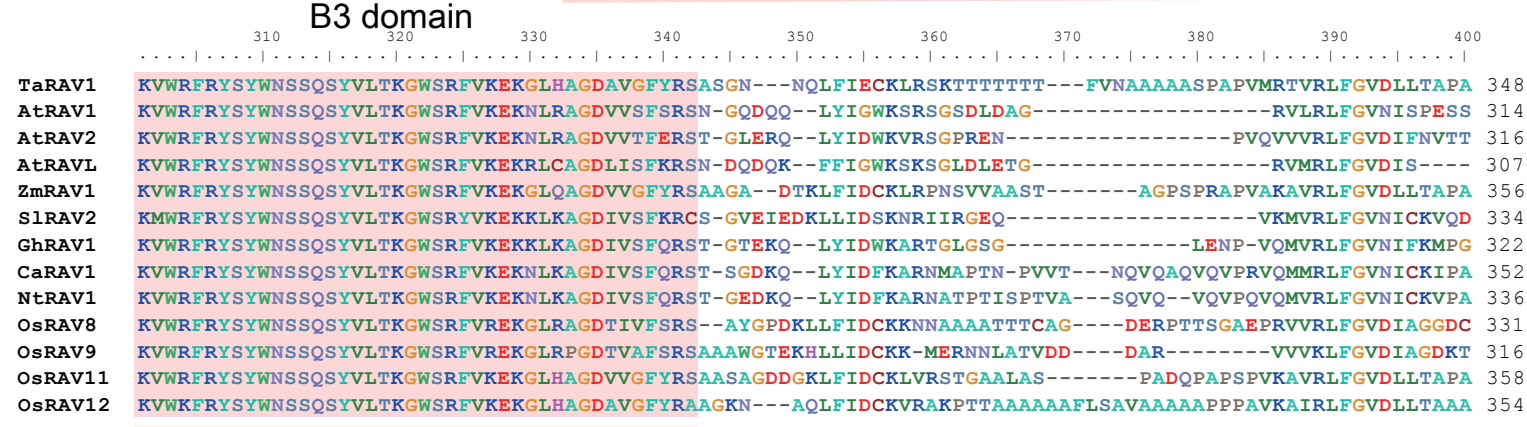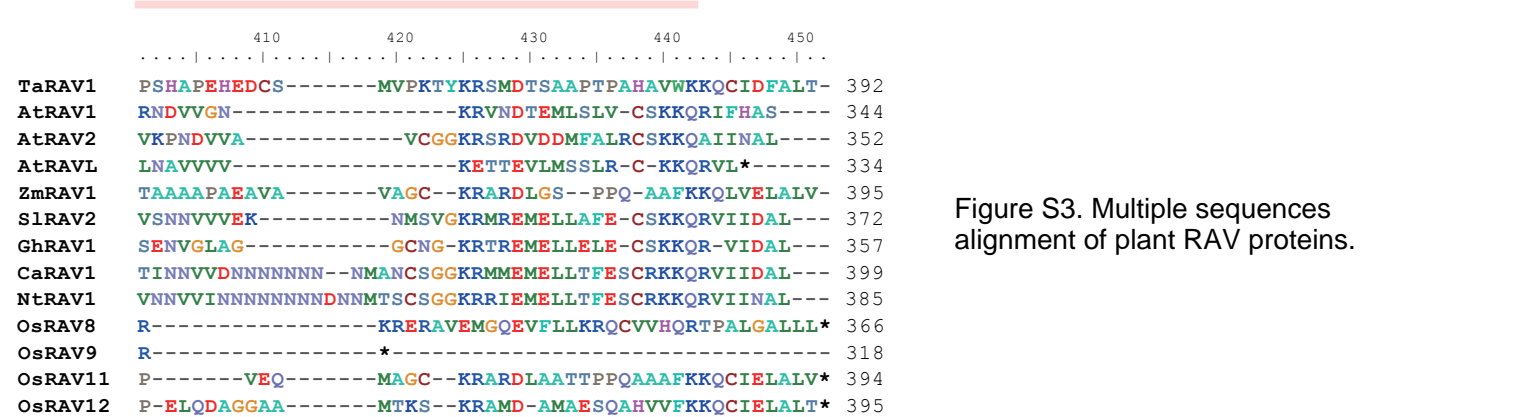

Figure S3. Multiple sequences alignment of plant RAV proteins.

Supplement: Supplementary file 1 [file ijms-23-08834-s001.zip › Figure S3.pdf]

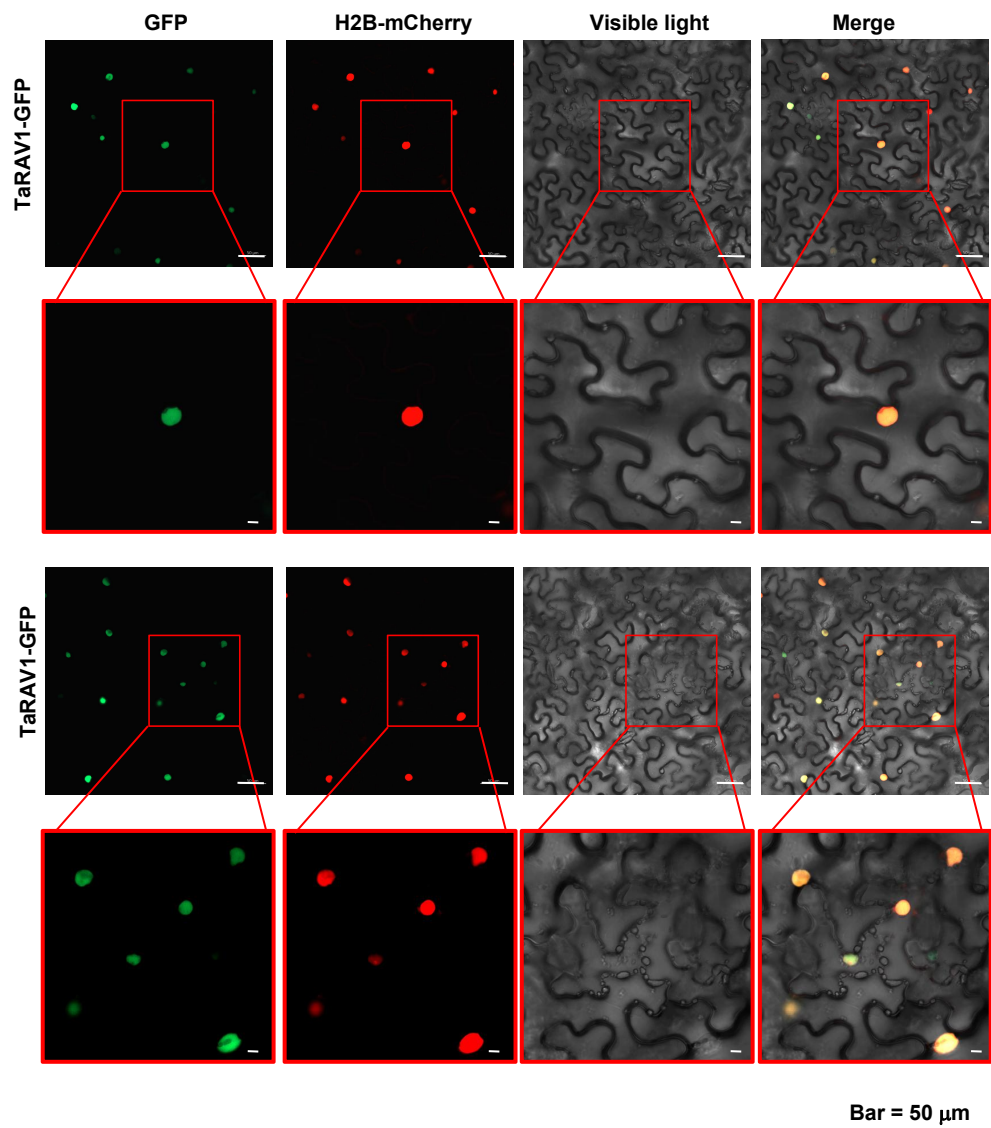

**Figure S5.** Subcellular localization of TaRAV1 protein.

Supplement: Supplementary file 1 [file ijms-23-08834-s001.zip › Figure S5.pdf]
